# The metabolic syndrome-insulin resistance index: a tool for identifying dyslipidemia across varied glucose metabolic score in patients with cardiovascular disease

**DOI:** 10.3389/fendo.2025.1473308

**Published:** 2025-05-12

**Authors:** Lu Yu, Yutong Liu, Ruiying Guo, Tong Yang, Guangwei Pan, Yuanyuan He, Shan Gao, Rongrong Yang, Zhu Li, Lin Li, Chunquan Yu

**Affiliations:** ^1^ Institute of Traditional Chinese Medicine, Tianjin University of Traditional Chinese Medicine, Tianjin, China; ^2^ School of Basic Medical Sciences, Zhejiang Chinese Medical University, Hangzhou, China

**Keywords:** METS-IR, insulin resistance, dyslipidemia, glucose metabolic states, cardiovascular disease

## Abstract

**Purpose:**

The METS-IR index, a non-insulin-based metabolic score, represents a new marker closely linked to insulin resistance. This study aimed to evaluate the relationship between the METS-IR index and dyslipidemia in individuals diagnosed with Cardiovascular disease (CVD), as well as to delve deeper into how varying glucose metabolic conditions influence this relationship.

**Methods:**

This multicenter retrospective investigation encompassed 214,717 individuals diagnosed with CVD across China, spanning from September 1, 2014, to June 1, 2022, ultimately incorporating 17,632 cases in the conclusive analysis. All cases were grouped according to quartiles of METS-IR. The American College of Cardiology classifies dyslipidemia into four distinct categories: hyper-triglyceridemia (hyper-TG), hyper-cholesterolemia (hyper-TC), hypo-high-density lipoprotein cholesterolemia (hypo-HDL), and hyper-low-density lipoprotein cholesterolemia (hyper-LDL). Dyslipidemia is diagnosed when any one of these conditions is present. Logistic regression analysis was performed to estimate the odds ratio (OR) and 95% confidence interval (CI), assessing the relationship between the METS-IR index and dyslipidemia risk in patients with CVD. To evaluate the precision of the METS-IR index in identifying dyslipidemia, receiver operating characteristic (ROC) curve was produced.

**Results:**

The results of the baseline analysis showed that 11,934 cases had dyslipidemia, with notable variations observed in the clinical and biological attributes among CVD cases (*P* < 0.05 to < 0.001). Logistic regression analysis showed that the METS-IR index was significantly associated with the risk of dyslipidemia (odds ratio [OR]: 1.14; 95% confidence interval [CI] 1.13-1.15; *P* < 0.001). The OR for dyslipidemia in Q4 of the METS-IR index was 11.94 (95% CI 10.60-13.45; *p* < 0.001) compared to Q1. ROC analysis revealing an area under the curve (AUC) of 0.747 (95% CI 0.739-0.754; *P* < 0.001). The association between the METS-IR index and dyslipidemia proved significant across all glycemic status groups, with the highest OR observed in the Q4 subgroup of cases with NGR (OR: 15.43; 95% CI 12.21-19.49).

**Conclusion:**

The risk of developing dyslipidemia is positively associated with heightened METS-IR levels in individuals afflicted with CVD, and these relationships hold significance across all glycemic metabolic conditions. METS-IR could potentially aid in forecasting the risk of dyslipidemia development in individuals diagnosed with CVD.

## Background

Dyslipidemia and Cardiovascular disease (CVD) are chronic conditions that significantly impact public health (1). Rapid socio-economic progress in recent decades has led to a substantial rise in the global burden of dyslipidemia (2). Dyslipidemia, a readily modifiable independent risk factor for CVD (3), can have its associated risks mitigated or postponed through early detection and prompt intervention (4).

Insulin resistance (IR) is considered to have a significant role in the development of dyslipidemia. Within the European Group for Insulin Resistance Research (EGIR), insulin sensitivity, as measured by the hyperinsulinemic-euglycemic clamp method, showed a strong correlation with triglyceride (TG) levels (5, 6). The intricate relationship between IR and alterations in lipid and lipoprotein metabolism contributes to atherosclerotic dyslipidemia, which is believed to elevate the risk of CVD (3).

To date, the established benchmark for evaluating IR is the hyperinsulinemic euglycemic clamp technique (HEC) (7). Conventional approaches to IR evaluation are constrained in terms of their real-world applicability, primarily as a result of their intricacy, invasiveness, and expense. The Metabolic Score for Insulin Resistance (METS-IR) Index is a recently devised indicator that exhibits a stronger correlation with HEC compared to other IR indices not based on insulin (8). However, there is a scarcity of research examining the relationship between METS-IR and dyslipidemia.

Consequently, the objective of this research was to examine the relationship between the METS-IR index and dyslipidemia in a substantial cohort of patients diagnosed with CVD, as well as to assess the impact of varying glucose metabolic conditions on this relationship. Furthermore, this research also determined the precision of the METS-IR index in identifying dyslipidemia among patients diagnosed with CVD.

## Methods

### Subjects

**Figure 1:**
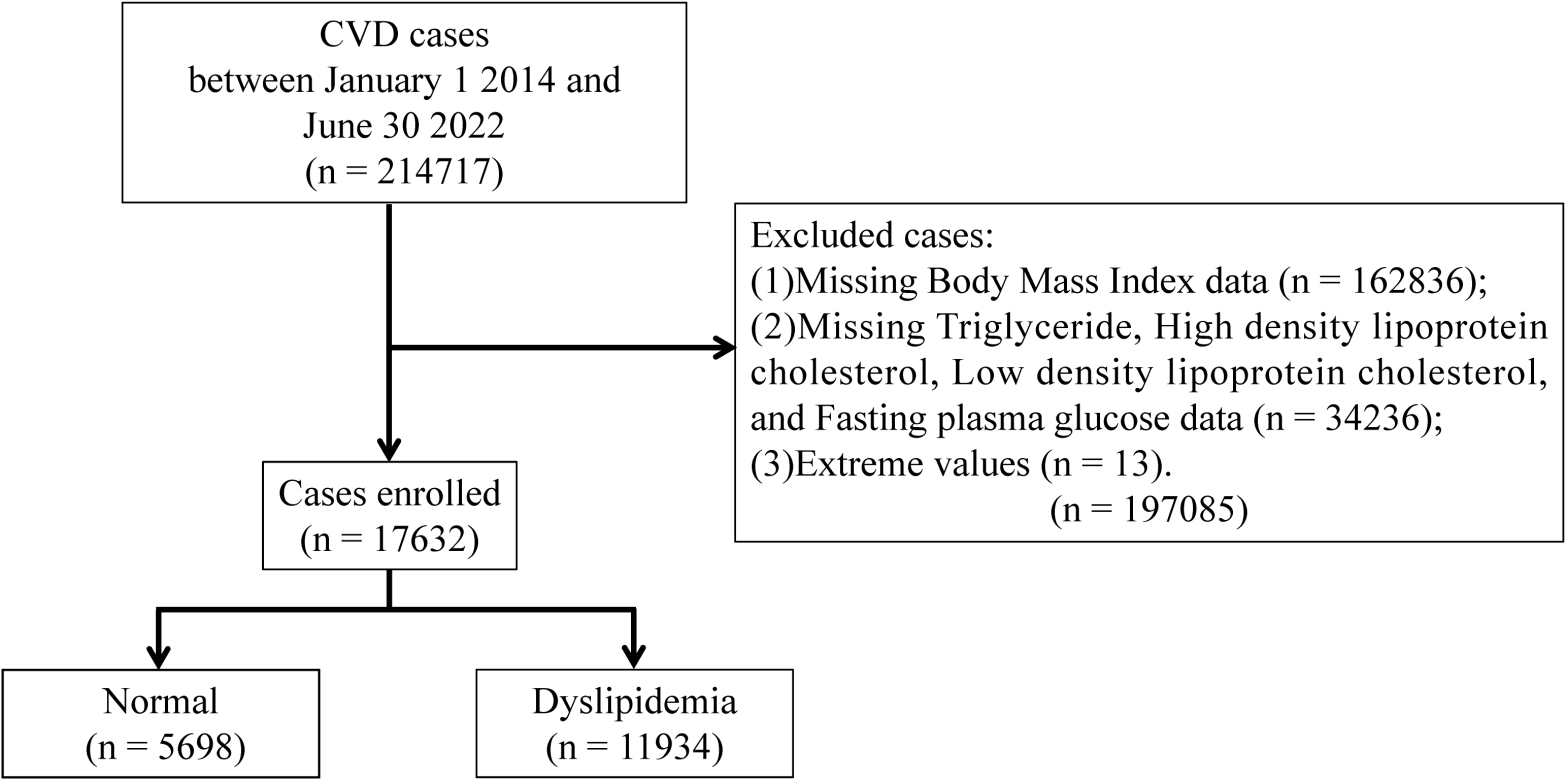
The flow chart of patient recruitment.

This large-scale multicenter retrospective study population comprised 214,717 CVD cases hospitalized in Tianjin between January 1, 2014, and June 30, 2022. The following cases were excluded: (1) those have extreme values; and (2) those who lacked data on Body Mass Index (BMI), TG, High Density Lipoprotein Cholesterol (HDL-C), Low Density Lipoprotein Cholesterol (LDL-C), and Fasting Plasma Glucose (FPG). Based on previous research, we estimated the overall probability as P≈68%, with an allowable error of δ≈0.15P, leading to a calculated sample size of 41 cases (9). To strengthen the robustness of our findings, we maximized the sample size. Ultimately, a total of 17,632 cases were included in the final analysis. The schematic representation illustrating the cases recruitment process is depicted in **Figure 1**. Approval for the study was obtained from the Ethics Committee of Tianjin University of Traditional Chinese Medicine (TJUTCM-EC20210007) and registered with the China Clinical Trials Registry (ChiCTR2200058296) and with Clinical Trials. gov (NCT05309343).

### Data collection

Age, sex, smoking, drinking, and medication history of cases were collected through standardized questionnaires, which were administered approximately four hours following their admission (10, 11). Systolic blood pressure (SBP), diastolic blood pressure (DBP), FPG, glycated hemoglobin (HbA1c), TC, TG, HDL-C, and LDL-C levels were recorded by practitioners using standardized laboratory methods (12).

### Definitions

METS-IR was calculated as (ln [(2 × FPG) + TG)] × BMI)/(ln [HDL-C]) (FPG, TG and HDL-C levels were expressed as mg/dl and BMI as kg/m^2^ in the equation) (13). BMI was calculated by dividing weight by square height and was expressed in kg/m^2^. Hyper-triglyceridemia (hyper-TG), hyper-cholestero lemia (hyper-TC), hyper-high-density lipoprotein cholesterolemia (hyper-HDL), hyper-low-density lipoprotein cholesterolemia (hyper-LDL) were defined as TG ≥ 1.7 mmol/L, TC ≥ 5.2 mmol/L, HDL-C < 1.0 mmol/L, and LDL-C ≥ 3.4 mmol/L, respectively, and dyslipidemia if any one of them (14). According to the American Diabetes Association’s Standards for the Medical Treatment of Diabetes (15), diabetes mellitus (DM) is defined as FPG ≥7.0 mmol/L or HbA1c ≥6.5%, prediabetes mellitus (Pre-DM) is defined as 5.6 mmol/L≤FPG ≤ 6.9 mmol/L or 5.7%≤HbA1c ≤ 6.4%, and normal glucose regulation (NGR) is defined as FPG<5.6 mmol/L or HbA1c<5.7%.

### Statistical analysis

Continuous data are presented as mean ± standard deviation (SD) or median and interquartile range (IQR). Categorical variables are expressed as percentages. Differences between the groups were calculated using the χ^2^ test for categorical variables and the t-test Mann-Whitney test or Kruskal-Wallis test for continuous variables. Odds ratios (OR) and 95% confidence intervals (CI) were calculated using logistic regression analysis to test the association between METS-IR index and the risk of dyslipidemia in cases with CVD. To ascertain the precision of the METS-IR index in identifying dyslipidemia among cases diagnosed with CVD, the area under the curve (AUC) was derived from the receiver operating characteristic (ROC) curve analysis. The statistical significance threshold was set at a *P*-value of less than 0.05. All statistical analyses were performed using the SPSS 24.0 (IBM Corp, New York, NY, USA).

## Results

### Clinical and biological characteristics

Among the 17,632 cases incorporated into this study, the proportion of males (55.75%) was slightly higher than that of females (44.2%); the median age of cases stood at 65 years, with 11,934 cases exhibiting dyslipidemia. Relative to the normolipidemic cohort, the dyslipidemic group predominantly comprised males, exhibited a lower mean age, registered higher METS-IR scores, and demonstrated a predisposition towards deleterious habits, including smoking and drinking (*P* < 0.01) (**Table 1**).

**Table 1:** Baseline characteristics.

Characteristic	Total (n=17632)	Normal (n=5698)	Dyslipidemia (n=11934)	*P*-value
Sex				< 0.001
Males	9830 (55.75)	2831 (49.68)	6999 (58.65)	
Females	7802 (44.25)	2867 (50.32)	4935 (41.35)	
Age (y)	65 (58,70)	67 (61,72)	64 (57,70)	< 0.001
FPG (mmol/L)	5.88 (5.17,7.40)	5.63 (5.05,6.79)	6.02 (5.23,7.70)	< 0.001
LDL-C (mmol/L)	2.44 (1.84,3.15)	2.20 (1.75,2.70)	2.62 (1.91,3.47)	< 0.001
HDL-C (mmol/L)	1.04 (0.88,1.22)	1.20 (1.09,1.36)	0.93 (0.82,1.10)	< 0.001
TG (mmol/L)	1.42 (1.04,1.99)	1.06 (0.83,1.31)	1.74 (1.25,2.30)	< 0.001
TC (mmol/L)	4.04 (3.32,4.88)	3.80 (3.28,4.35)	4.24 (3.34,5.22)	< 0.001
SBP (mmHg)	135 (124,149)	135 (124,148)	135 (123,149)	0.536
DBP (mmHg)	78 (70,86)	78 (70,85)	79 (71,87)	< 0.001
METS-IR index	41.20 (36.48,46.57)	37.19 (33.27,41.33)	43.30 (38.78,48.54)	< 0.001
Glucose metabolism state				
NGR	7316 (41.49)	2755 (48.35)	4561 (38.22)	< 0.001
Pre-DM	4914 (27.87)	1606 (28.19)	3308 (27.72)	< 0.001
DM	5402 (30.64)	1337 (23.46)	4065 (34.06)	< 0.001
Drinking	1996 (11.32)	585 (10.27)	1411 (11.82)	0.002
Smoking	2506 (14.21)	742 (13.02)	1764 (14.78)	0.002

Data are presented as median (interquartile) or number (proportion, %).

FPG, fasting plasma glucose; LDL-C, low-density lipoprotein cholesterol; HDL-C, high-density lipoprotein cholesterol; TG, triglycerides; TC, total cholesterol; SBP, systolic blood pressure; DBP, diastolic blood pressure; NGR, normal glucose regulation; Pre-DM, pre-diabetes; DM, diabetes.

### Association between METS-IR index and dyslipidemia

In the context of binary logistic regression analyses, continuous variables revealed a significant and positive association between the METS-IR index and the risk of developing dyslipidemia. Furthermore, the METS-IR index exhibited a positive association with an elevated OR for dyslipidemia in quartiles Q2, Q3, and Q4, with Q1 serving as the reference group. This association persisted as statistically significant even after adjusting for confounding variables. With ascending levels of the METS-IR index, the ORs for dyslipidemia were observed to be 2.34 (95% CI 2.15-2.55), 4.45 (95% CI 4.05-4.89), and 11.94 (95% CI 10.60-13.45), sequentially (all *P* < 0.001) (**Table 2**).

**Table 2:** Association between METS-IR index and dyslipidemia.

Variables	dyslipidemia					hyper-cholesterolemia (hyper-TG)				
	OR (95% CI)^a^	*P*-value	OR (95% CI)^b^		*P*-value	OR (95% CI)^a^	*P*-value	OR (95% CI)^b^		*P*-value
**METS-IR**	**1.14 (1.13-1.15)**	**<0.001**	**1.14(1.13-1.15)**		**<0.001**	**1.12(1.12-1.13)**	**< 0.001**	**1.12(1.12-1.13)**		**< 0.001**
Q1	Reference		Reference			Reference		Reference		
Q2	2.36(2.15-2.57)	<0.001	2.34(2.15-2.55)		<0.001	2.84(2.54-3.18)	<0.001	2.84(2.54-3.18)		<0.001
Q3	4.52(4.12-4.96)	<0.001	4.45(4.05-4.89)		<0.001	5.11(4.58-5.70)	<0.001	5.11(4.58-5.71)		<0.001
Q4	12.22(10.87-13.74)	<0.001	11.94(10.60-13.45)		<0.001	10.78(9.65-12.03)	<0.001	10.87(9.72-12.17)		<0.001
Variables	hyper-cholesterolemia (hyper-TC)					hyper-high-density lipoprotein cholesterolemia (hyper-HDL)				
	OR (95% CI)^a^	*P*-value	OR (95% CI)^b^		*P*-value	OR (95% CI)^a^	*P*-value	OR (95% CI)^b^		*P*-value
**METS-IR**	**1.00 (1.00-1.01)**	**1.00**	**1.00 (1.00-1.01)**		**0.30**	**1.00 (0.99-1.00)**	**0.20**	**1.00 (0.99-1.00)**		**0.21**
Q1	Reference		Reference			Reference		Reference		
Q2	0.95(0.85-1.06)	0.34	0.96(0.86-1.07)		0.45	1.13(1.01-1.26)	0.03	1.12(1.01-1.25)		0.04
Q3	0.94(0.84-1.05)	0.29	0.95(0.85-1.07)		0.42	1.11(0.99-1.24)	0.06	1.09(0.98-1.22)		0.11
Q4	1.03(0.92-1.16)	0.56	1.07(0.96-1.21)		0.23	1.02(0.91-1.14)	0.80	1.00(0.89-1.12)		0.98
Variables	hyper-low-density lipoprotein cholesterolemia (hyper-LDL)									
	OR (95% CI)^a^			*P*-value		OR (95% CI)^b^			*P*-value	
**METS-IR**	**1.85 (1.69-2.02)**			**<0.001**		**1.94 (1.77-2.12)**			**<0.001**	
Q1	Reference					Reference				
Q2	3.10(2.78-3.45)			<0.001		3.05(2.74-3.40)			<0.001	
Q3	6.69(6.01-7.44)			<0.001		6.54(5.88-7.29)			<0.001	
Q4	15.92(14.23-17.80)			<0.001		15.16(13.53-16.98)			<0.001	

a Model 1: adjusted for age, sex.

b Model 2: adjusted for age, sex, smoking, drinking, antihypertensive, antilipidemic, and antidiabetic drug therapy.

OR, odds ratio; CI, confidence interval; hyper-TG, hyper-cholesterolemia; hyper-TC, hyper-cholesterolemia; hyper-HDL, hyper-high-density lipoprotein cholesterolemia; hyper-LDL, hyper-low-density lipoprotein cholesterolemia; METS-IR, metabolic score for insulin resistance.

The METS-IR indice was divided into quartiles, Q1, METS-IR < 36.48; Q2, 36.48 ≤ METS-IR  ≤ 41.20; Q3, 41.20 ≤ METS-IR ≤  46.57; Q4, METS-IR  > 46.57.

Stratified analyses were performed in **Table 2**, which were conducted with consideration for the disparities among distinct subcategories of dyslipidemia. The METS-IR index exhibited a positive association with the risk of hyper-TG and hyper-LDL, albeit without significant association concerning the concurrent risks of hyper-TG and hyper-TC. The ORs corresponding to the METS-IR index in conjunction with hyper-TG and hyper-TC for quartiles two through four (Q2, Q3, and Q4), respectively, exhibited a persistent upward trend as the METS-IR value increased, with Q1 serving as the baseline comparator. Importantly, these observed associations sustained their statistical significance following the application of adjustments for a range of confounding variables (all *P* < 0.001).

**Figure 2:**
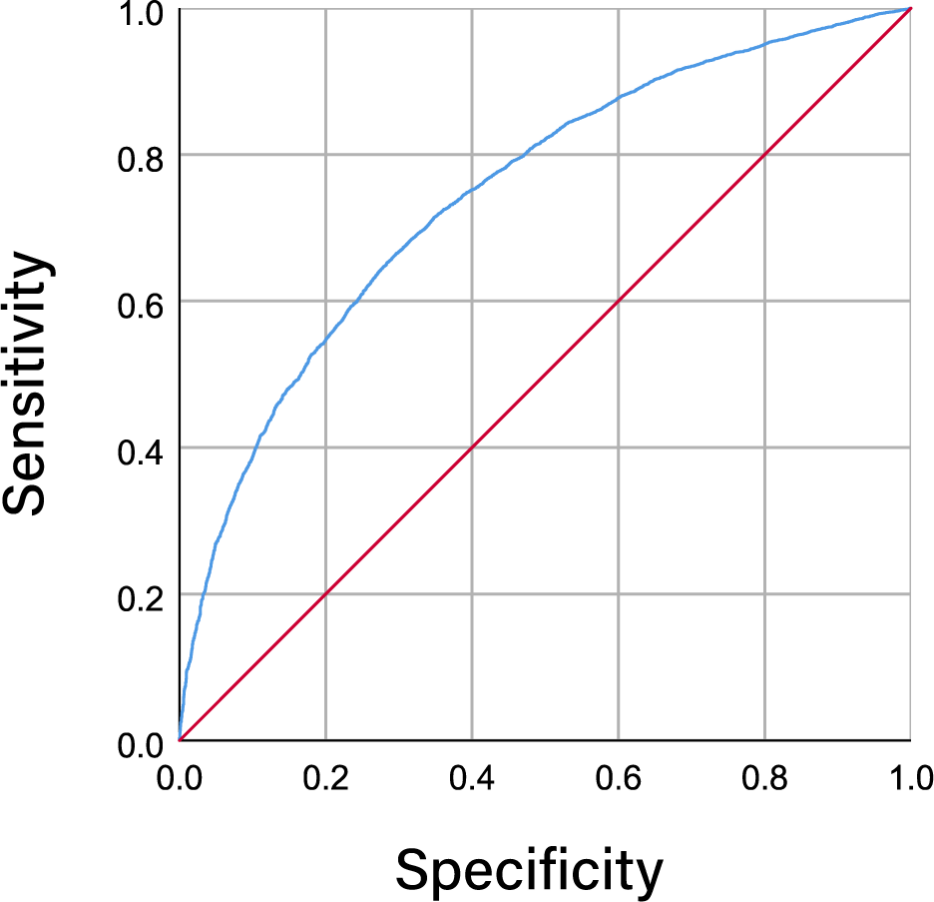
ROC curve for the use of METS-IR index in the detection of dyslipidemia.

**Figure 2** illustrates the ROC curves representing dyslipidemia and the METS-IR index among individuals diagnosed with CVD. The area under the curve (AUC) metric for the METS-IR index was calculated to be 0.747 (95% CI 0.739-0.754; *P* < 0.001), suggesting a statistically significant capacity of the METS-IR index in identifying and diagnosing dyslipidemic conditions.

### Associations between METS-IR index and dyslipidemia in different glucose metabolism states

The employment of binary logistic regression analysis served to evaluate the impact of varying glucose metabolic conditions on the association between the METS-IR index and the presence of dyslipidemia, with findings summarized in **Table 3**. Concerning the METS-IR index as a continuously measured variable, a statistically significant association was observed with the incidence of dyslipidemia across all distinct categories of glucose metabolic conditions (*P* < 0.001). Furthermore, in the context where Q1 served as the reference, the ORs associated with the presence of dyslipidemia were observed to escalate in conjunction with ascending levels of the METS-IR index, with Q4 having the highest OR (all *P* < 0.01). These associations persisted in their significance following the adjustment of the model parameters.

**Table 3:** Associations between METS-IR index and dyslipidemia in different glucose metabolism states.

Glucose metabolism state	Variables	Dyslipidemia			
		OR (95% CI)^a^	*P-*value	OR (95% CI)^b^	*P-*value
NGR	METS-IR index	1.15 (1.14-1.16)	< 0.001	1.15 (1.14-1.16)	< 0.001
	Q1	Reference		Reference	
	Q2	2.626 (2.328-2.961)	< 0.001	2.61 (2.31-2.95)	< 0.001
	Q3	5.123 (4.437-5.915)	< 0.001	5.09 (4.4-5.88)	< 0.001
	Q4	15.56 (12.328-19.64)	< 0.001	15.43 (12.21-19.49)	< 0.001
Pre-DM	METS-IR index	1.13 (1.12-1.15)	< 0.001	1.13 (1.12-1.15)	< 0.001
	Q1	Reference		Reference	
	Q2	2.12 (1.806-2.50)	< 0.001	2.11 (1.79-2.48)	< 0.001
	Q3	3.901 (3.278-4.642)	< 0.001	3.85 (3.24-4.59)	< 0.001
	Q4	10.34 (8.28-12.91)	< 0.001	10.29 (8.23-12.87)	< 0.001
DM	METS-IR index	1.13 (1.12-1.14)	< 0.001	1.13 (1.12-1.15)	< 0.001
	Q1	Reference		Reference	
	Q2	1.94 (1.59-2.37)	< 0.001	1.95 (1.60-2.39)	< 0.001
	Q3	3.82 (3.14-4.65)	< 0.001	3.89 (3.19-4.74)	< 0.001
	Q4	9.84 (7.96-12.17)	< 0.001	9.93 (8.01-12.31)	< 0.001

a Model 1: adjusted for age, sex.

b Model 2: adjusted for age, sex, smoking, drinking, antihypertensive, antilipidemic, and antidiabetic drug therapy.

## Discussion

This is the first study to reveal a significant association between the METS-IR index and dyslipidemia in cases with CVD and to assess this association according to different glucose metabolic states. In the present study, the METS-IR index was significantly associated with dyslipidemia, and this association was particularly significant in different quartiles of the METS-IR index. These associations can still be observed at different levels of glucose metabolism as well. The novelty of this study is to analyze the more convenient and reliable METS-IR index and its association with the risk of dyslipidemia, and directly relates the IR to lipid metabolism changes and advance current knowledge.

Previous observational studies have reported an association between glycolipid metabolism and increased risk of CVD (16, 17). Nuclear magnetic resonance analyses showed that the mean particle size of very low density lipoprotein (VLDL) was larger and the mean particle size of LDL and HDL was smaller in IR individuals compared to insulin-sensitive individuals (18). Besides, postprandial VLDL, Apo B-48, Apo B-100, cholesterol and TG concentrations are more elevated in patients with T2DM compared to their non-diabetic peers (19). Furthermore, A 13-year follow-up study reported that metabolic syndrome due to IR is a key risk factor for CVD, leading to approximately twice the mortality rate from CVD (20). A study by the EGIR found that the HOMA-IR index was strongly correlated with TG concentrations (5). These show a strong link between insulin and lipid levels in CVD patients. Our study supports this by more thoroughly demonstrating that the HOMA-IR index is associated with dyslipidemia in CVD patients.

There are several pathways that may explain the observed association between HOMA-IR index and dyslipidemia. Insulin resistance increases hepatic glucose output and conversely enhances triglyceride synthesis and VLDL release. Intracellular accumulation of lipids triggers activation of novel protein kinase C and subsequently leads to impaired insulin signaling. In the liver, insulin normally stimulates glucose uptake, glucose oxidation and glycogen synthesis, inhibits gluconeogenesis, and stimulates triglyceride synthesis and secretion via VLDL (21). IR is a generic term which implies that adipose tissue, skeletal muscle, liver and pancreas are less responsive to the action of insulin.IR increases hepatic TG synthesis, and increased TG synthesis is differentially correlated with increased hepatic Apo B-100 production (22, 23). This results in hypertriglyceridemia, a variable increase in the number of particles reflected by VLDL apolipoprotein B-100, and a decrease in HDL-C concentration (24). Furthermore, IR is also associated with elevated hepatic triglyceride lipase (HTGL), which may lead to accelerated HDL clearance and abnormal HDL-C (25).

A variety of lipids such as TG and TC, as well as HDL and LDL, are involved in the regulation of microvascular function, and dyslipidemia reduces coronary flow reserve and capillary density, induces apoptosis of capillary endothelial cells, and ultimately leads to impaired left ventricular (LV) function (26), exacerbating the condition of CVD patients. In addition, dyslipidemia leads to myocardial ultrastructural alterations through a variety of mechanisms. High fat and high cholesterol (HFHC) diets increase serum TC and free fatty acid (FFA) levels, leading to systemic oxidative stress and a pro-inflammatory state, which in turn leads to apoptosis and cardiac injury (27, 28). Long-term prospective epidemiological studies have consistently shown that the incidence of coronary heart disease is significantly lower in individuals with a favorable lipid profile (29). Prevention and rational management of dyslipidemia can significantly alter cardiovascular morbidity and mortality.

The pathogenesis of dyslipidemia includes IR, hyperinsulinemia and abnormal levels of adipokines, with IR being a prominent feature of the metabolic syndrome and T2DM-type diabetes mellitus, which also leads to the development of CVD (30). Several mechanisms by which IR exacerbates atherosclerosis have been elucidated, including systemic inflammation, endothelial dysfunction and oxidative stress (31). The HEC technique is the gold standard for the assessment of IR, while HOMA-IR is the most widely used method due to its technically costly and time-consuming nature. All this explains why the comprehensive monitoring index of METS-IR is useful in developing strategies for primary prevention and management of dyslipidemia in patients with CVD.

### Limitations

The current study has several limitations. First, although we accounted for numerous potential confounding factors, dyslipidemia may still be influenced by other variables, such as systemic lupus erythematosus, hereditary dyslipidemia, or cardiac catheterization. Second, this study was conducted in a Chinese population, where racial differences may exist. Third, potential Neyman bias should be considered. Therefore, well-designed randomized controlled trials are needed to validate these results.

## Conclusion

The risk of developing dyslipidemia is associated with elevated levels of METS-IR in individuals with coronary artery disease, and this correlation is significant across glucose metabolism states. METS-IR may help predict the risk of developing dyslipidemia in individuals with coronary artery disease.

## Data Availability

The raw data supporting the conclusions of this article will be made available by the authors, without undue reservation.
